# BAP1: case report and insight into a novel tumor suppressor

**DOI:** 10.1186/s12895-017-0065-6

**Published:** 2017-11-22

**Authors:** Kanad Ghosh, Badri Modi, William D. James, Brian C. Capell

**Affiliations:** 1 Penn Epigenetics Institute, University of Pennsylvania Perelman School of Medicine, Philadelphia, Pennsylvania 19104 USA; 2 Department of Dermatology, University of Pennsylvania Perelman School of Medicine, Philadelphia, Pennsylvania 19104 USA; 3 Biomedical Research Building 1007, University of Pennsylvania, Philadelphia, Pennsylvania 19104 USA

**Keywords:** BAP1, Tumor suppression, Familial cancer syndrome

## Abstract

**Background:**

BRCA1-Associated-Protein 1 (BAP1) is a dynamic tumor suppressor which, when mutated, has been associated with an increased risk of uveal melanoma, cutaneous melanoma, mesothelioma, and several other cancers. Germline *BAP1* mutations have been extensively studied, where they have been found to cause hereditary cancer susceptibility. However, their sporadic counterparts, tumors that display a loss of BAP1 expression due to somatically arising mutations in the *BAP1* gene, remain a poorly described entity.

**Case presentation:**

Here we present the case of a 49-year-old female who presented with an asymptomatic dome-shaped pink papule on the dorsal foot which was found on biopsy to be deficient in the BAP1 tumor suppressor. While the patient’s family history did not suggest the presence of a familial cancer syndrome, germline genetic testing was performed and was negative. The patient underwent surgical excision of this sporadically appearing “BAPoma” by Mohs surgery.

**Conclusions:**

Given the relatively banal clinical appearance of these dome-shaped neoplasms, sporadic BAPomas may often be overlooked by clinicians and dermatologists. In addition to providing a representative case, here we also provide a synopsis of the current understanding of these neoplasms, both in terms of the histopathological features, as well as the molecular mechanisms underlying BAP1 function and its ability to prevent tumorigenesis.

## Background

Within the last decade, the BRCA1-Associated-Protein 1 (BAP1) has been increasingly appreciated for its tumor suppressor activities, given that a loss of BAP1 can drive carcinogenesis in diverse tissue types. Germline mutations in *BAP1* have been described in families with a hereditary increase in the risk of uveal melanoma, cutaneous melanoma, mesothelioma, Merkel cell carcinoma, and several other cancers [1]. Strikingly, a large-scale systematic review found that patients with *BAP1* mutations face increased mortality, both general and cancer-specific, as well as increased likelihood of cancer relapse [2]. Thus, mutations in BAP1, along with family history, can be used as an assessment for a patient’s risk of certain cancers, and confers importance to the knowledge of a patient’s *BAP1* mutation status.

More recently, sporadic somatic *BAP1* mutations have been shown to occur in the setting of both mesothelioma and uveal melanoma [3]. In the skin, various neoplasms deficient in BAP1 have been described, typically presenting as dome-shaped to pedunculated growths which range from pink to light brown in color [1, 4]. These BAP1 negative tumors, or “BAPomas”, can be screened for using immunohistological staining, following which genetic testing can confirm the presence or absence of a *BAP1* mutation in the germline. Sporadic BAPomas have been less thoroughly studied and reported than their familial counterparts. We present the case of a patient who presented with a sporadic BAPoma, describe her management, and provide a brief update on the current understanding of BAPoma histopathological categorization and BAP1 function.

## Case presentation

**Fig. 1:**
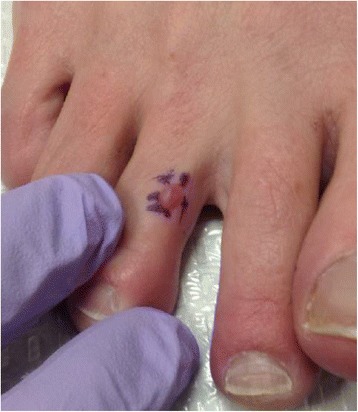
Clinical image of the patient’s red-purple dome-shaped papule on the right dorsal third toe

A 49-year-old female presented with an asymptomatic pink to purple dome-shaped papule on the third digit of her right foot (Fig. 1). She has a history of actinic keratoses, but otherwise no other dermatological issues. Her family history is significant for a history of colon cancer and prostate cancer, as well as hypertension, hyperlipidemia, coronary artery disease, and diabetes, but no history of breast or ovarian cancer. A shave biopsy was performed.

**Fig. 2:**
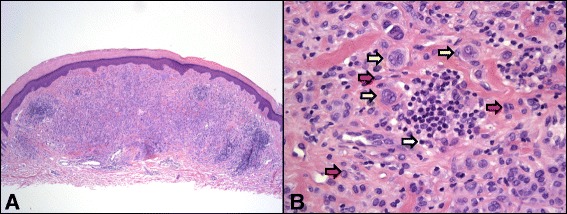
Histopathology (10×) demonstrated a predominantly dermal melanocytic proliferation (**a**). It consisted of nests and strands of atypical epithelioid melanocytes (yellow arrows in **b**) surrounded by clusters of lymphocytes (white arrow in **b**) and pigmented macrophages (red arrows in **b**)

**Fig. 3:**
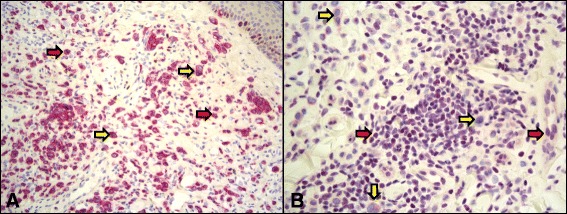
MART-1 staining (**a**) demonstrates positive staining of both typical (red arrows) and atypical melanocytes (yellow arrows). **b** BAP1 staining does not highlight the nuclei of atypical cells (yellow arrows), but does stain typical melanocytes (red arrows), suggesting the atypical epithelioid melanocytes have lost BAP1 expression

The sample proved to be a primarily dermal melanocytic proliferation (Fig. 2a), with a low proliferative index as determined by Ki67 staining. The lesion contained nests and strands of atypical epithelioid melanocytes surrounded by lymphocytes and pigmented macrophages (Fig. 2b). The lesion stained positive for MART-1 (Fig. 3a), however, BAP1 staining was negative (Fig. 3b), leading to the diagnosis of a BAPoma with epithelioid atypia and rare mitoses. Given that the atypical cells extended to the margins of the biopsy, a re-excision was recommended and the patient underwent Mohs surgery to clear the lesion. Although her personal and family history was not suggestive of a BAP1-associated cancer syndrome, the patient underwent genetic testing for a germline mutation in the *BAP1* gene. Sequencing and deletion/duplication analysis of the *BAP1* gene was negative, evidence supporting a sporadic *BAP1* mutation in the lesion where the BAPoma formed. As there are no clear guidelines on screening for other cancers such as renal cell carcinoma or mesothelioma in these patients, only continued screening for cutaneous was recommended.

## Discussion

Prior to the discovery of its key tumor suppressor role, BAP1 was primarily known for its role as a deubiquitinase. Ubiquitination is a common post translational modification by which proteins can be marked for degradation, with ubiquitin ligases and deubiquitinase working in opposition to maintain quality control of cellular proteins. In addition its clear role in ubiquitination and maintaining protein balance, BAP1 has been shown to be critical for a range of cellular processes associated with cancer, including differentiation and DNA repair [5, 6]. In addition, BAP1 has been implicated in the DNA damage response through its involvement in the ataxia telangiectasia-mutated (ATM) signaling pathway, as well as in epigenetic transcriptional regulation associated with preventing cancerous proliferation [7–9].

This basic knowledge has helped unravel BAP1’s role in tumor suppression in vivo. The 90 kDa protein was initially thought to reduce tumorigenesis through the deubiquitination of BRCA1, though it was later revealed that BAP1 can achieve its tumor suppressive role independently of BRCA1 [10]. Mechanistically, BAP1 localizes to the nucleus and employs its deubiquitinase activity to promote G1/S cell cycle transition and the induction of cell death, likely through impairment of normal DNA repair processes [10, 11]. Consistent with this, lung cancer cells with mutant BAP1 typically possess truncations or other mutations that negatively impact its deubiquitinase and nuclear localization ability. Most recently, extensive mechanistic work has demonstrated that BAP1-deficient cells not only accumulate increased levels of DNA damage, but also are unable to undergo apoptosis as compared to similarly damaged cells with normal levels of BAP1 [12]. In melanocytes, which undergo less turnover than neighboring keratinocytes, this accumulated damage likely combines with a failure of DNA repair and apoptosis to the increased chance of neoplastic proliferation seen both in patients with both inherited and sporadic deficiency of BAP1.

Histopathologically, cutaneous BAPomas are usually intradermal, comprised of a majority epithelioid melanocytes and containing large amounts of amphophilic cytosol with well-distinguished boundaries [4]. Some authors have classified these sporadic tumors into the heterogeneous of umbrella of atypical Spitz tumors, given that spitzoid melanocytic neoplasms can display immunohistochemical BAP1 staining that may be positive, negative, or only perinuclear with absent nuclear staining [13]. However, positive BRAFV600E expression in many BAP1 negative lesions, as well as a lack of epidermal hyperplasia, clefting between melanocytes, and Kamino bodies, sets this population apart from traditional Spitz nevi [4, 14]. Indeed, the study of both sporadic and germline BAPomas has and will continue to provide new insights into a previously unknown tumor suppressive pathway in the skin.

## Abbreviations

ATM: Ataxia telangiectasia-mutated
BAP1: BRCA1-Associated-Protein 1
